# Clinical Characteristics and Treatment Outcomes of 65 Patients With BRAF-Mutated Non-small Cell Lung Cancer

**DOI:** 10.3389/fonc.2020.00603

**Published:** 2020-04-28

**Authors:** Yuxin Mu, Ke Yang, Xuezhi Hao, Yan Wang, Lin Wang, Yutao Liu, Lin Lin, Junling Li, Puyuan Xing

**Affiliations:** ^1^Department of Medical Oncology, National Cancer Center/National Clinical Research Center for Cancer/Cancer Hospital, Chinese Academy of Medical Sciences and Peking Union Medical College, Beijing, China; ^2^Department of Medical Oncology, Cancer Hospital of HuanXing, Beijing, China

**Keywords:** BRAF mutation, chemotherapy, immunotherapy, non-small cell lung cancer, targeted therapy

## Abstract

BRAF mutation is an oncogenic driver gene in non-small cell lung cancer (NSCLC) with low frequency. The data of patients with NSCLC harboring BRAF mutations is rare. We conducted a retrospective multicenter study in Chinese patients with NSCLC harboring BRAF mutations between Jan 2017 and Jul 2019. A total of 65 patients treated in 22 centers were included, 54 harbored BRAF-V600E mutation and 11 had non-V600E mutations, including K601E, G469S, G469V, G469A, G596R, G466R, and T599dup. Of 18 patients with early-stage disease at diagnosis and underwent a resection, the median disease-free survival (DFS) was 43.2, 18.7, and 10.1 months of stage I, II, and IIIA patients, respectively. In 46 patients with advanced-stage disease at data cutoff, disease control rate (DCR), and progression-free survival (PFS) of first-line anti-BRAF targeted therapy was superior than chemotherapy in patients harboring BRAF-V600E mutation (DCR, 100.0 vs. 70.0%, *P* = 0.027; median PFS, 9.8 vs. 5.4 months, *P* = 0.149). Of 30 V600E-mutated patients who received anti-BRAF therapy during the course of disease, median PFS of vemurafenib, dabrafenib, and dabrafenib plus trametinib was 7.8, 5.8, and 6.0 months, respectively (*P* = 0.970). Median PFS were similar between V600E and non-V600E patients (5.4 vs. 5.4 months, *P* = 0.825) to first-line chemotherapy. Nine patients were treated with checkpoint inhibitors, with median PFS of 3.0 months. Our data demonstrated the clinical benefit of anti-BRAF targeted therapy in Chinese NSCLC patients harboring BRAF-V600E mutation. The value of immunotherapy and treatment selection among non-V600E population needs further study.

## Introduction

Lung cancer is one of the most common cancers and remains the leading cause of cancer-related death worldwide (1). The successful applying of epidermal growth factor receptor (EGFR) tyrosine kinase inhibitor (TKI) in non-small cell lung cancer (NSCLC) patients who harbored EGFR mutations has dramatically changed the therapeutic approach of lung cancer and led to a more individualized treatment era. Patients with oncogenic driver mutations may benefited in driver gene inhibitors rather than cytotoxic chemotherapy. V-raf murine sarcoma viral oncogene homolog B1 (BRAF) mutation is one of oncogenic driver mutation in NSCLC, which phosphorylates the downstream effectors MEK and ERK to promote cell proliferation and survival (2). BRAF mutations occur with a low prevalence of only 2–5% in Caucasian lung cancers, and V600 mutations (amino acid substitution for valine at position 600) accounted for ~50%, with the rest of cases harbor non-V600 mutations (3–5). BRAF inhibitors (BRAFi) and MEK inhibitors (MEKi) have been demonstrated impressive efficacy in patients with advanced stage NSCLC harboring BRAF V600E mutation. Monotherapy BRAFi vemurafenib showed an objective response rate (ORR) of 43% in patients with refractory BRAF V600E-mutated NSCLC in the “MyPathyway” basket study (6). In an open-label, phase 2 trial, BRAFi dabrafenib plus MEKi trametinib performed an ORR of 64%, and median progression-free survival (PFS) of 10.9 months (95% confidence interval [CI] 7.0–16.6) in patients with previously untreated BRAF V600E-mutant metastatic NSCLC (7). Unlike the definite and promising efficacy of targeted therapies to BRAF V600E-mutant cases, the benefit of targeted agents on various non-V600 mutations were questionable, as each specific non-V600 mutation occurred in a much smaller population thus there were few studies on this topic. In EURAF cohort, five of six patients who harbored non-V600E mutations appeared to be resistant to BRAFi therapies (8). The prevalence of BRAF mutation was even lower in Chinese NSCLC patients with reported of 0.5–2% (9, 10). Considering the difference in genetic background between Caucasians and Asians, studying the BRAF mutation of NSCLC in Asians is of great significance. Clinical efficacy of chemotherapy and targeted therapy in Chinese patients with NSCLC harboring BRAF mutations are not well-explored due to their low prevalence, especially for those with non-V600 mutations, thus none of BRAFi has been approved for BRAF-mutated NSCLC in China. In addition, BRAFi plus MEKi was theoretically efficient in patients progressed of BRAFi monotherapy, but with fewer actual clinical data. Moreover, immune checkpoint inhibitors were increasingly used in clinical practice in China as monotherapy or in combination with chemotherapy, while its efficacy in BRAF-mutated patients is still an unmet area. Therefore, we performed this retrospective study to evaluate the association of BRAF mutations with clinical characteristics and treatment outcomes in Chinese NSCLC patients in the real-world.

## Materials and Methods

### Patient Recruitment and Data Collection

Patients were retrospectively recruited through a patient community. Potential subjects could contact study recruiter individually for more details about the study and eligibility screening. The inclusion criteria included (i) patients were histologically or cytologically diagnosed with NSCLC and were detected harboring BRAF mutation between Jan 2017 and Jul 2019. (ii) BRAF mutation was detected using a next-generation sequencing (NGS) technique, which also provided molecular profile of EGFR, KRAS, ALK, MET, ROS1, HER2, RET, PIK3CA, and NTRK status as well. Patients with a BRAF mutation that never received a treatment for stage IV disease were also included in our study for baseline characteristics analysis. Patients who tested positive for EGFR, ALK, MET, ROS1, or RET, and those who acquired BRAF mutation after resistance to therapies targeting another oncogenic driver gene were ineligible. After receiving study subjects' oral consent, qualified patients were asked to provide their medical records for data collection. To ensure the quality of study data, all medical data were reviewed, and entered by a board-certified oncologist with thoracic expertise from Cancer Hospital of Chinese Academy of Medical Sciences. By the end of July 2019, a total of 65 NSCLC patients with BRAF mutation treated in 22 hospitals in China were included in our analysis. Medical data of age, gender, smoking history, Eastern Cooperative Oncology Group performance status (ECOG PS), histology, stage, BRAF mutation type, and treatment history were retrospectively recorded. Age, smoking status, and ECOG PS were recorded at initial diagnosis. Stage of disease was determined according to the American Joint Committee on Cancer (AJCC) staging system, 8th edition. The study was approved by the Ethics Committee at Cancer Hospital of Chinese Academy of Medical Sciences, and was conducted in accordance with the Helsinki Declaration.

### Assessments

The primary objective of our study was to evaluate the efficacy of chemotherapy, anti-BRAF targeted therapy, and immunotherapy in patients with BRAF-mutated NSCLC. The primary endpoints were disease control rate (DCR) and PFS. Tumor response was evaluated according to the Response Evaluation Criteria in Solid Tumors version 1.1 (RECIST v1.1). DCR was defined as the percentage of patients who achieved complete response (CR), partial response (PR), or stable disease (SD), while ORR referred to CR and PR. PFS was defined as the time from the date of a systemic treatment regimen (chemotherapy, targeted therapy, or immunotherapy) initiation till date of progressive disease (PD) or death from any causes whichever occurred first. Secondary endpoints were DFS of patients who was diagnosed with early-stage disease at initiation and safety profile of anti-BRAF targeted therapy. DFS was measured from the date of resection to recurrent or metastases.

### Statistical Analysis

The distribution of patients' baseline characteristics was described. Difference of ORR and DCR between groups were compared using Fisher's exact tests and chi-square tests. Survival analysis was performed using the Kaplan–Meier method, and compared by the log rank test. Two-sided *p* < 0.05 was indicated statistically significant. All statistical analysis was carried out using the SPSS statistical software, version 23.0 (SPSS, Inc., Chicago, IL, USA).

## Results

### Patients Characteristics

A total of 65 patients with BRAF mutation were included in our study. All patients were Chinese, 31 were male and 34 were female with a median age of 58 (range, 33–79). Thirty-five patients (53.8%) were former or current smokers. Most patients had ECOG PS of 0 or 1 (86.2%) and stage IIIB to IV disease (46/65, 70.8%) at diagnosis. Sixty-four were adenocarcinomas and one was squamous cell carcinoma. In 18 early-stage patients who underwent pulmonary surgery, micropapillary component was observed in five patients (27.8%), and these micropapillary feature was only observed in V600E mutated patients. Patient characteristics are summarized in Table 1.

**Table 1:** Baseline characteristics of BRAF mutated NSCLC patients (*n* = 65).

**Characteristics**	**No. of patients (%)**		
	**All *N* = 65**	**V600E *n* = 54**	**Non-V600E *n* = 11**
**Age, years**			
Median	58	57.5	58
Range	33–79	33–78	46–79
**Sex**			
Male	31 (47.7)	23 (42.6)	8 (72.7)
Female	34 (52.3)	31 (57.4)	3 (27.3)
**ECOG PS**			
0–1	56 (86.2)	49 (90.7)	7 (63.6)
≥2	9 (13.8)	5 (9.3)	4 (36.4)
**Smoking status**			
Non-smoker	30 (46.2)	28 (51.9)	2 (18.2)
Former/current smoker	35 (53.8)	26 (48.1)	9 (81.8)
**Histology**			
Adenocarcinoma	64 (98.5)	53 (98.1)	11 (100.0)
Others	1 (1.5)	1 (1.9)	0 (0.0)
**Stage at diagnosis**			
0	1 (1.5)	0 (0.0)	1 (9.1)
I	10 (15.4)	9 (16.7)	1 (9.1)
II	5 (7.7)	4 (7.4)	1 (9.1)
IIIA	3 (4.6)	3 (5.6)	0 (0.0)
IIIB-IV	46 (70.8)	38 (70.4)	8 (72.7)
**Co-occurring mutation**			
TP53	4	2	2
PIK3CA	6	6	0
KRAS	1	0	1
NTRK1	1	1	0

Eight BRAF mutation genotypes were identified, 54 patients had BRAF-V600E mutation (83.1%) and 11 (16.9%) had non-V600E mutations, including K601E (6.2%, *n* = 4), G469S (1.5%, *n* = 1), G469V (1.5%, *n* = 1), G469A (1.5%, *n* = 1), G596R (1.5%, *n* = 1), G466R (1.5%, *n* = 1), and T599dup (3.1%, *n* = 2). Nine of 54 patients with a BRAF-V600E mutation had concomitant mutation in TP53 (*n* = 2), PIK3CA (*n* = 6) or NTRK1 (*n* = 1), and concurrent TP53 (*n* = 2) or KRAS mutation (*n* = 1) were identified in 3 of 11 patients with BRAF non-V600E mutations (Table 1). The frequency of co-alterations was similar in BRAF-V600E mutated patients and in non-V600E mutated population (16.7 vs. 27.3%, *P* = 0.689).

Eleven patients harbored non-V600E mutations, with median age of 58. Twenty-three (42.6%) of 54 BRAF-V600E patients and 8 of 11 (72.7%) non-V600E patients were male, respectively (*P* = 0.068). Twenty-six (48.1%) of 54 BRAF-V600E patients and 9 of 11 (81.8%) non-V600E patients were smokers, respectively (*P* = 0.041). There was no significant difference in age and histology distribution between patients with BRAF-V600E and non-V600E mutations.

### Clinical Outcomes

#### DFS in Early-Stage Patients

**Figure 1:**
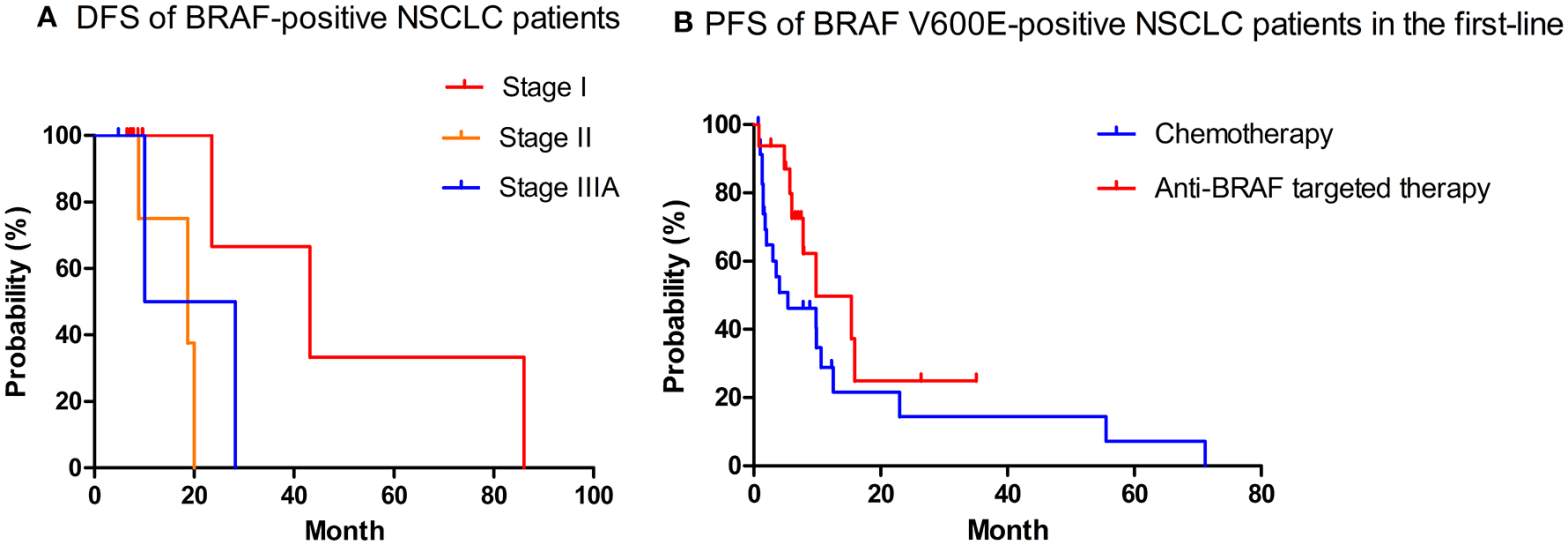
DFS of early-stage BRAF-positive NSCLC patients **(A)**, PFS of first-line regimens in patients with BRAF-positive NSCLC **(B)**. DFS, disease-free survival; PFS, progression-free survival. Tick marks indicate censored observations.

Among overall 65 patients in our study, 1 was stage 0, 10 were stage I, 5 were stage II, 3 were stage IIIA, and 46 were advanced stage (IIIB-IV) at diagnosis, the median follow-up time was 9.2 months. At data cutoff (Jul 31, 2019), 8 of 18 recurrences (44.4%) had occurred in patients who had early-stage disease at diagnosis and underwent a resection, among whom seven had distant metastasis while only one performed locoregional recurrence. The site of relapse included lung (*n* = 2), brain (*n* = 2), bone (*n* = 2), mediastinal lymph nodes (*n* = 2), supraclavicular lymph nodes (*n* = 2), pleura (*n* = 1), and adrenal gland (*n* = 1). The median DFS after surgery of early-stage cancers was 43.2 months of stage I, 18.7 months of stage II, and 10.1 months of stage IIIA patients (*P* = 0.07), respectively (Figure 1A). One patient with stage II disease was excluded as he did not undergo resection.

#### Clinical Outcomes of First-Line Treatment

In 46 patients with advanced stage BRAF-V600E mutated NSCLC at data cutoff, 25 patients received chemotherapy in the first-line (19 with pemetrexed-contained regimen, 5 with paclitaxel-contained regimen, 1 with gemcitabine-contained regimen), while only 16 patients received anti-BRAF targeted therapy as the first-line choice (9 with vemurafenib, 2 with dabrafenib, 5 with dabrafenib plus trametinib). Twenty and 15 patients were evaluable for response analysis in chemotherapy and targeted therapy subgroups, respectively. Of patients who received chemotherapy in response analysis set, 5 patients had PR, 9 had SD, and 6 had PD, with ORR of 25.0%. Among patients treated with targeted therapy, 10 patients had PR, 5 had SD, and ORR was 66.7%. DCR of first-line targeted therapy was higher than that of chemotherapy in patients with BRAF-V600E mutated NSCLC (100.0 vs. 70.0%, *P* = 0.027). The median PFS of patients with BRAF-V600E mutation who received first-line targeted therapy was also longer than chemotherapy, but the difference did not achieve statistical significance (9.8 months [95%CI, 0.4, 19.2] vs. 5.4 months [95%CI, 0.0, 14.1], *P* = 0.149) (Figure 1B).

**Figure 2:**
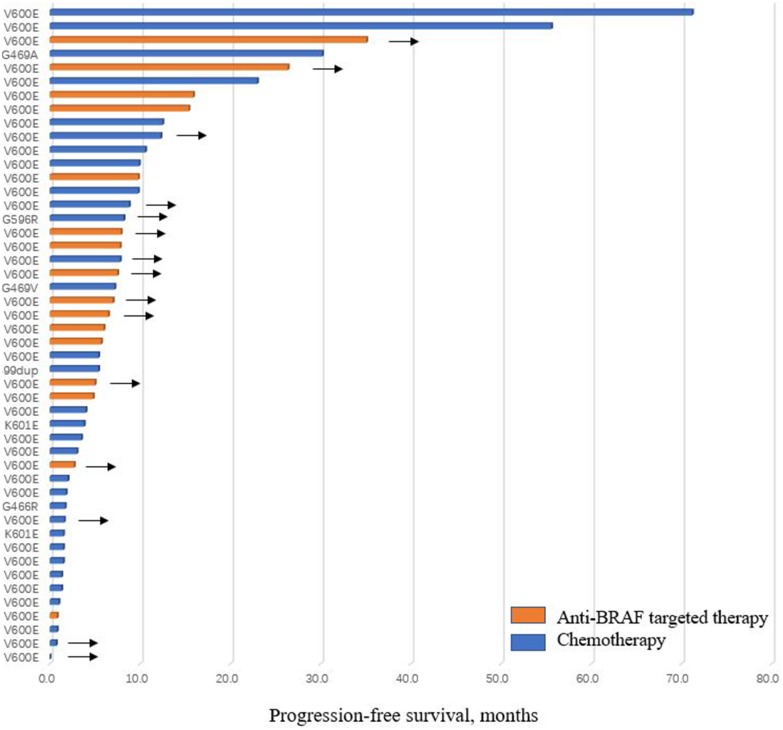
Progression-free survival of patients treated with chemotherapy or anti-BRAF targeted therapy in the first-line. Arrows indicate patients did not progress at last follow-up.

Within BRAF non-V600E subgroup, pemetrexed-contained regimen was the most widely used first-line treatment regimen (7/9, 77.8%). Five of seven (71.4%) measurable patients had SD, and 2 had PD. None of them received targeted therapy in the first-line. No significant differences of ORR and DCR were observed in patients with V600E and non-V600E mutation who were treated with first-line chemotherapy (ORR, 25.0 vs. 0.0%, *P* = 0.283; DCR, 70.0 vs. 71.4%, *P* = 1.000). The median PFS of first-line chemotherapy was also similar between patients with V600E mutation vs. those with non-V600E mutation (5.4 months [95%CI, 0.0, 14.1] vs. 5.4 months [95%CI, 1.3, 9.5], *P* = 0.825). The efficacy of first-line regimens in patients with BRAF mutated advanced NSCLC was shown in Table 2, Figure 2.

**Table 2:** Efficacy of first-line treatment strategies in patients with BRAF mutation.

**Treatment strategies in first-line**	**V600E**		**Non-V600E**	
	**DCR**	**PFS months, (95%CI)**	**DCR**	**PFS months, (95%CI)**
Pemetrexed-contained chemotherapy	11/14, 78.6%	5.4 (1.7, 9.1)	5/7, 71.4%	5.4 (1.3, 9.5)
Paclitaxel-contained chemotherapy	2/5, 40.0%	1.5 (1.1, 1.9)	–	–
Vemurafenib	9/9, 100.0%	9.8 (0.7, 18.9)	–	–
Dabrafenib	1/1, 100.0%	–	–	–
Dabrafenib + Trametinib	5/5, 100.0%	NR	–	–

*DCR, disease control rate; PFS, progression-free survival; NR, not reached*.

For patients who performed multiple mutations, patients with co-occurring mutations in TP53 had a trend of shorter PFS of first-line treatment compared with those without TP53 mutation (median PFS, 3.5 months [95%CI, 0.5, 6.5] vs. 9.8 months [95%CI, 4.9, 14.7], *P* = 0.106). Median PFS of patients with co-occurring PIK3CA mutations was 12.5 months (95%CI, 5.4, 19.6), as compared to 7.2 months (95%CI, 3.7, 10.7) in patients without PIK3CA mutation (*P* = 0.823).

#### Targeted Therapy

Thirty-two of the 55 patients with advanced stage BRAF mutated NSCLC cases were treated with anti-BRAF targeted therapy during their treatment course, among whom 30 harbored V600E mutation, 1 harbored K601E mutation and 1 harbored T599dup. The only 2 non-V600E mutated patients received dabrafenib plus trametinib after failure of pemetrexed-platinum based chemotherapy and the T599dup case performed SD while the K601E patient had PD as the best response. In 30 patients with V600E mutation, 17 patients received BRAF inhibitor as first-line treatment and 13 had anti-BRAF therapy in further lines. Thirteen patients received vemurafenib, 6 patients received dabrafenib and 9 were treated by a combination of dabrafenib and trametinib as the primary targeted therapy. The median PFS of patients receiving vemurafenib, dabrafenib, and dabrafenib plus trametinib was 7.8, 5.8, and 6.0 months, respectively (*P* = 0.970) (Table 3). Five patients received two different targeted regimens, including four patients treated with vemurafenib followed by dabrafenib plus trametinib, and one patient treated with dabrafenib followed by dabrafenib plus trametinib. Efficacy of BRAFi plus MEKi after the failure of BRAFi monotherapy was generally very poor. Four of five patients showed PD, aside from 1 had a SD of dabrafenib plus trametinib after vemurafenib, with PFS of only 2.9 months.

**Table 3:** Efficacy of primary targeted therapy in patients with BRAF V600E mutation.

	**Vemurafenib**	**Dabrafenib**	**Dabrafenib + Trametinib**
First-line	9	2	5
Further-line	4	4	4
Evaluable for response analysis	13	5	9
DCR	12/13, 92.3%	5/5, 100.0%	9/9, 100.0%
PFS, months (95% CI)	7.8 (3.9, 11.7)	5.8 (0.2, 11.4)	6.0 (5.0, 7.0)

*DCR, disease control rate; PFS, progression-free survival*.

The safety analysis was conducted in patients who received anti-BRAF targeted therapy in the treatment course. For patients treated with vemurafenib, the most common adverse events (AEs) were arthralgia and rash. Four events of grade 3 AEs were observed, including arthralgia, rash and hand-foot syndrome. Dose reductions or interruptions of vemurafenib occurred in 6 (46.2%) patients. AEs of dabrafenib observed including fatigue, pyrexia, rash, mucositis oral, and anemia. One (16.7%) of 6 patients had AEs that led to dabrafenib dose reduction and subsequent dose interruption (grade 2 pyrexia and grade 3 rash). The most common AE among patients receiving dabrafenib plus trametinib regimen was pyrexia, and 4 (36.4%) patients had AEs that led to dose reductions or interruptions. No anti-BRAF targeted therapy-related deaths was observed in our study. AEs of each targeted regimen were shown in Table 4.

**Table 4:** Adverse events of targeted therapy.

**Type of AE**	**AE Grade**	**Vemurafenib *N* = 13**	**Dabrafenib *N* = 6**	**Dabrafenib + Trametinib *N* = 11**
Pyrexia	1	2	0	4
	2	1	1	0
	3	0	0	1
Arthralgia	1	4	0	1
	2	1	0	1
	3	2	0	1
Rash	1	5	0	1
	2	1	0	0
	3	1	1	0
Hand-foot syndrome	1	1	0	0
	2	2	0	1
	3	1	0	0
Fatigue	1	4	2	1
	2	1	0	1
Pneumonitis	1	0	0	0
	2	0	0	2
Loss of appetite	1	1	0	1
Mucositis oral	1	0	1	0
Nausea	1	2	0	0
Alopecia	1	3	0	0
ALT increased	1	1	0	0
White blood cell decreased	1	1	0	0
Anemia	1	0	1	0
Diarrhea	1	0	0	1

*AE, adverse events; ALT, glutamate pyruvic transaminase*.

#### Immunotherapy

Nine patients were treated with checkpoint inhibitors monotherapy or in combination with chemotherapy or anti-angiogenic treatment (6 of V600E, 3 of non-V600E). Two (25.0%) of 8 patients with measurable disease by RECIST 1.1 had PR, 3 (37.5%) had SD, and 3 (37.5%) had PD. Seven (77.8%) patients progressed on immunotherapy by the time of the analysis. Median PFS was 3.0 months (95%CI 2.9, 3.1). The 2 patients with PR had PFS of 8.9 and 3.0 months, respectively. From the 3 patients with non-V600E, one with K601E had SD with nivolumab plus chemotherapy, while the other two with T599dup and G466R had PD with pembrolizumab and nivolumab plus anlotinib, respectively (Table 5). Seventeen of 65 patients tested programmed death ligand 1 (PD-L1) or tumor mutational burden (TMB) during the course of disease, among whom six received checkpoint inhibitors. Considering the various antibody and NGS panel used for PD-L1 and TMB testing of study patients, the relation of these biomarkers, BRAF mutation and treatment efficacy was not analyzed.

**Table 5:** Characteristics of BRAF-mutated patients treated with immunotherapy.

**Patient**	**BRAF mutation**	**Regimen**	**Treatment line**	**Tumor response**	**PFS (months)**	**Status at last follow-up**
1	V600E	Pembrolizumab	2	PR	8.9	PR
2	K601E	Nivolumab + Chemotherapy	2	SD	3.5	SD
3	V600E	Nivolumab	1	Not measurable	3.0	PD
4	V600E	Nivolumab + Targeted therapy	3	SD	4.1	PD
5	V600E	Pembrolizumab + Bevacizumab	3	PR	3.0	PD
6	V600E	Nivolumab	2	PD	2.6	PD
7	T599dup	Pembrolizumab	2	PD	2.7	PD
8	V600E	Pembrolizumab + Targeted therapy	3	SD	5.5	PD
9	G466R	Nivolumab + Anlotinib	2	PD	2.0	PD

*PR, partial response; SD, stable disease; PD, progressive disease; PFS, progression-free survival*.

## Discussion

BRAF mutation was well-reported in papillary thyroid cancer, colorectal cancer, and melanoma, but not NSCLC in Chinese population due to its low prevalence. Some studies have reported the clinical and pathologic characteristics of NSCLC patients harboring BRAF mutations, our study mainly explored the treatment pattern and clinical outcomes of various BRAF genomic subtype among these patients.

BRAF mutation occurred in 0.5–2% of Chinese NSCLC patients (9, 10), which was lower than 2–5% in Caucasian lung cancers (3, 5, 11, 12). Our results showed that BRAF mutations are mostly performed in adenocarcinoma. The prevalence of BRAF-V600E mutations is 83.1%, which was consistent with previously reported in Chinese patients (10), while higher than Caucasian population of ~50% (3, 4, 13, 14). BRAF-V600E and non-V600E are associated with different clinical and pathologic features in our study. BRAF non-V600E mutations were more likely to be smokers and male, while V600E mutation occurred roughly equal both in gender and in smoking status, and micropapillary component was only observed in V600E-mutated population. The clinical features of gender and smoking status among BRAF-mutated NSCLC were different between studies. BRAF mutations in an Australian study occurred all in former smokers (5). Marchetti et al. suggested a significant predominance of female or never-smokers in patients harbored BRAF-V600E mutations and non-V600E mutations in smokers (3), while these studies were mostly focused on white patients. In Chinese studies, Ding et al. showed that BRAF mutations are more likely in never smokers (10), which is similar to patients with EGFR mutations. The discrepancy between studies may due to low sample size of BRAF-mutated NSCLC cases in each study and the difference of distribution of BRAF mutation subtypes between Caucasian and Asian. As for pathologic feature, a majority of BRAF-mutated NSCLC were adenocarcinomas, other histologic type such as squamous cell carcinoma and NSCLC, not otherwise specified (NOS) were also detected (5, 11, 14). The aggressive micropapillary component was a distinctive histologic feature showed partly in BRAF-V600E tumors, and in some studies, was independently associated with poor prognosis (3, 15).

Twelve (18.5%) patients harboring concurrent mutations were observed in our study, including TP53, PIK3CA, NTRK1, and KRAS mutations. The co-occurring rate among patients with BRAF-mutated NSCLC was reported as 14–16% (10, 12). Claire Tissot reported BRAF non-V600E mutations were associated with KRAS mutations in five cases who were all smokers, and suggested the concomitant KRAS mutation may be related to the carcinologic effect of tobacco (14). Whether the cooccurrence of KRAS mutation will impact response to targeted therapies is worthy of further exploration. Villaruz et al. suggested that patients with multiple mutations have inferior OS compared with those harbored single BRAF mutations (12). Additionally, it has been reported that tumors harboring TP53 mutations is associated with aggressive disease profile and worse clinical outcomes (16, 17), we also found that patients with coexisting TP53 mutation had shorter PFS of first-line treatment than those without a TP53 mutation, although the difference did not reach statistical significance. Unfortunately, due to limited cases with coexisting TP53, the clinical implications on treatment selection of such patients was not performed.

In our study, we evaluated the DFS of BRAF-mutated patients with early-stage radically resected NSCLC. Marchetti et al. reported BRAF V600E mutation was associated with a significantly shorter DFS and OS as compared to BRAF wild-type cases, suggesting a negative prognostic factor of BRAF-V600E mutation in early-stage NSCLC patients (3). Cardarella et al. also demonstrated a shorter DFS for BRAF V600-positive resected patients, while no difference between wild-type and mutation positive was observed in advanced-stage patients (11). Litvak et al. further showed that V600 mutant lung cancers performed an improved OS than non-V600 mutant cases in advanced-stage setting (13). The comparisons between studies should be made with caution as the discrepancy of baseline demographic characteristics between studies and the increasing treatment strategies as the development of medical oncology.

Chemotherapy and targeted therapy were two basic treatment strategies to BRAF-mutated patients. Cardarella et al. demonstrated a similar results of platinum-based combination chemotherapy between patients with BRAF mutation and those with wild type cancers (11). PFS of first-line pemetrexed-contained chemotherapy was equal in V600E and non-V600E subgroups in our analysis, while several studies (11) observed that response rate and PFS of platinum-based combination chemotherapy appeared a trend of favoring non-V600E population which may be attributed to the micropapillary histology of BRAF V600E-mutated population. We did not explore the association between micropapillary component and clinical outcomes considering the small sample size.

Anti-BRAF targeted therapy is the primary treatment for V600E-mutated cancers. In the NSCLC cohort of a basket study, vemurafenib achieved the ORR of 42% and median PFS of 7.3 months (95% CI, 3.5–10.8) among BRAF V600E–positive pre-treated NSCLC patients (18). The multicenter retrospective EURAF cohort explored the efficacy of known BRAF inhibitors (vemurafenib, dabrafenib, or sorafenib) in BRAF-mutated lung cancer. The median PFS and OS were 5.0 and 10.8 months, respectively, for overall anti-BRAF therapy (8). Dabrafenib was assessed in 78 pre-treated BRAF-V600E NSCLC patients, the ORR and DCR were 33% and 58%, respectively (19). BRAFi combining MEKi has proved to be more effective than single-agents for BRAF V600E-mutated lung cancers. Dabrafenib plus trametinib showed an ORR of 64% and median PFS of 10.9 months in patients with previously untreated BRAF V600E-mutant metastatic NSCLC in a phase 2 trial (7). To our knowledge, because of the low incidence rate of BRAF-mutated NSCLC in China, the efficacy and safety of anti-BRAF targeted therapy among BRAF-mutated Chinese NSCLC population in the real-world clinical practice remains unclear, the results of our study was of clinical importance. The results of our study were similar to that in clinical trials, which was superior to chemotherapy. AEs of targeted therapy were common in our study and performed diverse among patients. Arthralgia and rash were commonly observed in patients receiving vemurafenib, while pyrexia was frequently observed in dabrafenib monotherapy, or dabrafenib plus trametinib. Although it was not uncommon for patients receiving targeted therapy required dose reduction or interruption, most patients continued the doses and no severe AE was observed. Considering the superior efficacy and acceptable toxicity, anti-BRAF therapy was a better choice of first-line treatment for patients with BRAF-V600E mutated NSCLC. However, due to the limited sample size and the lack of head-to-head comparison, the specific choice among targeted agents in Chinese population was still an unmet area and could be guided by patient comorbidity and tolerability. For non-V600E mutation, the efficacy of single anti-BRAF targeted agent remains questionable. Non-V600E mutation was demonstrated lack of activity against BRAFi in clinical practice. The EURAF study (8) included six patients with non-V600E mutations, except for one harboring the G596V achieved PR with vemurafenib, the others (G466V, G469A, G469L, V600K, and K601E mutation) did not respond to BRAF inhibitors. Therefore, none patient with non-V600E mutation in our study received targeted therapy in the first-line. As non-V600E proportion in China was obviously lower than Caucasian, exploring optimal treatment strategy for such patients is even more difficult. Large-scale clinical exploration of diverse treatment strategies in this setting is warranted.

Immunotherapy is another treatment option emerging for patients with NSCLC, whereas the correlation between BRAF mutation and efficacy of immunotherapy is still unclear. Dudnik et al. (20) reported that the expression of PD-L1 was slightly higher in BRAF-mutant NSCLC than unselected population of previously reported, and a higher TMB in BRAF mutated patients was also observed. The median PFS of immunotherapy on BRAF V600E and non-V600E mutated patients was 3.7 and 4.1 months, respectively. The results seemed similar with unselected NSCLC (21, 22). Mazieres et al. (23) demonstrated that median PFS of immune checkpoint inhibitors monotherapy was significantly higher in smokers vs. never smokers (4.1 vs. 1.9 months, *P* = 0.03). In our study, a minority of patients tested PD-L1 or TMB, thus the relation of these biomarkers, BRAF mutation and treatment efficacy was not analyzed. As targeted therapy showed limited efficacy on non-V600E mutations, except for chemotherapy, investigating the efficacy of immunotherapy is highly in needed. We listed the outcomes of checkpoint inhibitors monotherapy or in combination in our patients, only 1 harboring K601E achieved SD to immunotherapy, the other 2 (T599dup and G466R) performed no response. Due to the limited number of cases, however, the findings need to be careful interpretation. Further researches of larger sample on Chinese population are needed to assess the efficacy of immunotherapy in BRAF-mutated cases.

As a retrospective study, several limitations to our analysis should be acknowledged. Study patients were retrospectively recruited through a patients community, thus a potential of selection bias may be introduced. Additionally, we lack the independent radiological review committee to re-evaluate treatment outcomes from diverse medical centers, and thus we used DCR, not ORR as our primary endpoint. Considering the heterogeneity of the follow-up, the interpretation of the results should be carefully illuminated. Furthermore, the small number of cases with BRAF non-V600E mutations limited the ability to draw conclusions on treatment selection and the power of interpretation to our outcomes. A multicenter, prospective study among Chinese patients harboring BRAF mutation in a larger cohort is needed.

In conclusion, our study confirmed the discrepancy of clinicopathological characteristics in BRAF mutated NSCLC among Chinese population. Anti-BRAF targeted therapy is more effective than chemotherapy, with manageable toxicity among BRAF-V600E mutated Chinese patients in the first-line setting. Chemotherapy was still the dominant treatment strategy for non-V600E population, and the place of immunotherapy for these patients needs further studies.

## Data Availability Statement

The raw data supporting the conclusions of this article will be made available by emailing corresponding authors.

## Ethics Statement

The studies involving human participants were reviewed and approved by National Cancer Center/National Clinical Research Center for Cancer/Cancer Hospital, Chinese Academy of Medical Sciences and Peking Union Medical College. Written informed consent for participation was not required for this study in accordance with the national legislation and the institutional approval.

## Author Contributions

JL and PX designed the study. YM collected, analyzed and interpreted the data, and prepared the manuscript. KY, XH, YW, LW, YL, and LL collected the data. All authors read and approved the final manuscript.

## Conflict of Interest

The authors declare that the research was conducted in the absence of any commercial or financial relationships that could be construed as a potential conflict of interest.
